# Intensity-Based Camera Setup for Refractometric and Biomolecular Sensing with a Photonic Crystal Microfluidic Chip

**DOI:** 10.3390/bios13070687

**Published:** 2023-06-27

**Authors:** Fabio Aldo Kraft, Stefanie Lehmann, Carmela Di Maria, Leonie Joksch, Stefanie Fitschen-Östern, Sabine Fuchs, Francesco Dell’Olio, Martina Gerken

**Affiliations:** 1 Integrated Systems and Photonics, Faculty of Engineering, Kiel University, 24118 Kiel, Germany; fkr@tf.uni-kiel.de (F.A.K.);; 2 Kiel Nano, Surface and Interface Science KiNSIS, Kiel University, 24118 Kiel, Germany; 3 Department of Electrical and Information Engineering, Polytechnic University of Bari, 70126 Bari, Italy; 4 Experimental Trauma Surgery, Department of Trauma Surgery and Orthopedics, University Medical Center Schleswig-Holstein, Kiel University, 24105 Kiel, Germany

**Keywords:** photonic crystal slab, label-free, point-of-care, microfluidics, biochip

## Abstract

Label-free sensing is a promising approach for point-of-care testing devices. Among optical transducers, photonic crystal slabs (PCSs) have positioned themselves as an inexpensive yet versatile platform for label-free biosensing. A spectral resonance shift is observed upon biomolecular binding to the functionalized surface. Commonly, a PCS is read out by a spectrometer. Alternatively, the spectral shift may be translated into an intensity change by tailoring the system response. Intensity-based camera setups (IBCS) are of interest as they mitigate the need for postprocessing, enable spatial sampling, and have moderate hardware requirements. However, they exhibit modest performance compared with spectrometric approaches. Here, we show an increase of the sensitivity and limit of detection (LOD) of an IBCS by employing a sharp-edged cut-off filter to optimize the system response. We report an increase of the LOD from (7.1 ± 1.3) × 10^−4^ RIU to (3.2 ± 0.7) × 10^−5^ RIU. We discuss the influence of the region of interest (ROI) size on the achievable LOD. We fabricated a biochip by combining a microfluidic and a PCS and demonstrated autonomous transport. We analyzed the performance via refractive index steps and the biosensing ability via diluted glutathione S-transferase (GST) antibodies (1:250). In addition, we illustrate the speed of detection and demonstrate the advantage of the additional spatial information by detecting streptavidin (2.9 µg/mL). Finally, we present the detection of immunoglobulin G (IgG) from whole blood as a possible basis for point-of-care devices.

## 1. Introduction

Label-free biosensing is a promising platform for inexpensive and easily available point-of-care (POC) devices [1]. Due to the absence of labeling, the handling is greatly simplified, which enables use by untrained personnel. Furthermore, the quick availability of specific panels of biomarkers is of great importance for the assessment of a patient’s health. For instance, Cardenas [2] has shown that assessment of the thrombin levels of trauma patients helps in predicting the development of serious complications during their hospital stay. In addition, Apple et al. [3] have shown that checking troponin levels of emergency room patients suffering from myocardial infarction reduces their length of stay in hospital. Moreover, the epidemic of SARS-CoV-2 has shown how quickly the demand for easy-to-use and readily available point-of-care testing devices might emerge [4]. Zanchetta et al. [5] pointed out in their review that label-free sensing allows detection of the binding behavior in real time and potentially enables the detection of small molecules, as occluding molecules due to labelling are omitted. Finally, label-free platforms are versatile, enabling a wide range of applications.

Label-free sensing has been studied on platforms such as electrical, electrochemical, mass-sensitive, or optical transducers [6]. In this work, we focus on optical transducers. Label-free sensing with optical transducers has been demonstrated with surface plasmon resonance (SPR) [7], ring resonators [8,9], slot waveguides [10,11], and photonic crystal slabs (PCS) [12,13,14]. Here, we focus on PCS.

A PCS is a nanostructured waveguide commonly processed on a solid substrate such as glass. Upon illumination, the nanostructure acts as a grating coupler and diffracts light that satisfies the Bragg equation into the waveguide. The light propagates along the waveguide as a quasi-guided mode. Due to the grating, light is coupled back out and leads to constructive and destructive interferences in reflection and transmission, respectively. This leads to guided-mode resonances (GMR) in the spectrum [15,16,17]. The behavior is described by

*mλ*_GMR_ = Λ (*n*_eff_ ± sin (α)), (1)

where *λ*_GMR_ is the wavelength of the guided-mode resonance, Λ is the period length of the nanostructure, *n*_eff_ is the effective refractive index of the mode, α is the angle of incidence of the light, and *m* is the Bragg mode integer [18,19]. We analyze first-order behavior and set *m* = 1. The electrical field distribution of the guided mode is not confined to the boundaries of the waveguide [20,21]. It has exponential components, which decay into the superstrate and substrate. These fields are called evanescent waves and extend approximately 50 nm to 100 nm out of the waveguides [22,23,24]. This renders the PCS sensitive to changes of the refractive index at the surface. Any change of refractive index leads to a shift of the guided-mode resonance of the PCS, as shown in Equation (1).

Different options exist to track resonance changes. A common approach is based on spectrometric readout [25,26,27,28]. While a spectrometer enables a chromatic resolution, it is a rather bulky instrument and a significant cost factor for point-of-care applications. A compact alternative is based on a photodetector or camera-based readout as was shown by Lin et al. [29], Kenaan et al. [30], and Jahns et al. [31,32]. Lin et al. [29] and Jahns et al. [31] used the system response of their setups to translate the chromatic shift into an observable change of intensity. In this case, the system response is the light emitting diode (LED) spectrum and the GMR lies on the falling shoulders of the LED. Any change in resonance position leads to a change of intensity and is thus detectable by camera or photodiode. However, they reported moderate limits of detection (LOD) in the range of 1.48 × 10^−4^ RIU [29] to 3.36 × 10^−4^ RIU [32]. The limit of detection is defined as the triple noise level divided by the sensitivity. It is commonly estimated by the expression

LOD = 3σ/S,(2)

where σ is standard deviation and S is the sensitivity of the setup.

One main factor influencing the achievable LOD is the quality factor Q. It is defined as the resonance wavelength divided by the full width at half maximum (FWHM) [33,34]. The higher the Q factor, the smaller the resonance width, which simplifies the detection of small changes in the resonance wavelength [35,36]. For PCS, the Q factor is reduced with the depth of the grating grooves of the nanostructure and the refractive index contrast. The Q factor is also a measure of stored energy [37]. This common framework was recently expanded by Conteduca et al. [38] and they introduced the importance of amplitude with regards to the achievable LOD. They argue that the LOD is optimized not merely by improving the Q factor, but by balancing the Q factor and the amplitude of the resonance. In addition, Drayton et al. [39] discuss noise-based limitations when using a PCS-based sensor. They review related scientific research and highlight that the best reported LOD achieved with PCS were in the range of 10^−6^–10^−5^ RIU. Yet, they note that these were often reached in a controlled environment with expensive equipment. They introduced a compact setup, using a camera, an LED, and a narrowband filter and were able to achieve an LOD of (5.8 ± 1.7) × 10^−5^ RIU. However, their signal detection depends upon peak fitting. Recently, Li et al. [40] have shown that the choice of fitting algorithm strongly influences the achievable LOD. The need for postprocessing for data evaluation should be minimized for any point-of-care application, as it influences the performance and introduces additional steps.

In this work we expand on the knowledge of an intensity-based camera setup (IBCS) to detect biomarkers on the basis of PCS. We improve the achievable LOD by employing a sharp-edged cut-off filter for narrowband sampling. We highlight the underlying concept and evaluate the effect the filter has on the obtained LOD. Furthermore, we demonstrate that the size of the regions of interest (ROI) strongly influences the achievable LOD in an IBCS. As a concept for a possible single-use microfluidic biochip, we report the binding of glutathione S-transferase (GST) antibodies to immobilized GST protein as well as streptavidin to immobilized bovine serum albumin (BSA) conjugated with biotin from a buffered solution. In addition, we report the binding of immunoglobulin G (IgG) to immobilized protein A/G from whole blood within a microfluidic biochip for direct sensing from unpurified whole blood.

This paper is structured as follows. In Section 2 we introduce the materials and methods used. In Section 3 the obtained results are shown and discussed as they are presented. In Section 4 we draw conclusions from the presented work.

## 2. Materials and Methods

### 2.1. Intensity-Based Camera Setup (IBCS)

**Figure 1:**
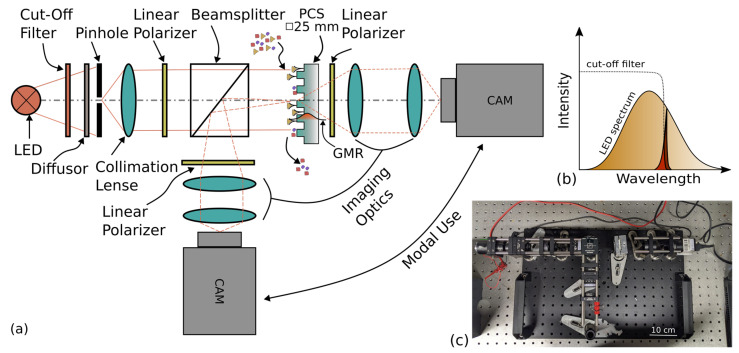
Schematic, working principle, and photograph of the setup. (**a**) Schematic of the intensity-based camera setup (IBCS). The detection is employable in transmission as well as reflection. The PCS size is square with a lateral dimension of 25 mm. The guided-mode resonance (GMR) has an evanescent field, which extends into the superstrate of the photonic crystal slab (PCS), enabling refractive index sensing. (**b**) Underlying working principle of the IBCS. The photonic crystal slab exhibits a guided-mode resonance when illuminated. The excitation light is suppressed due to the orthogonal polarizer setup. Upon binding, the resonance shifts. Here, this shift is transduced into an intensity shift, as the resonance lies on the falling edge of the system response. The setup is enhanced by a cut-off filter which is positioned in the setup. Its falling edge overlaps with the wavelength of the GMR. (**c**) Photograph of the setup.

The schematic of the setup is shown in Figure 1a. We employ an orthogonally crossed polarizers approach [31,41,42,43]. Embedding the PCS between two orthogonally crossed polarizers suppresses background light not coupling to the PCS [44,45]. The PCS was placed at an angle of 45° with respect to the two polarizing axes of the polarizers. This enables coupling of transverse magnetic (TM) and transverse electric (TE) modes to the PCS. Any non-coupled light is suppressed once it hits the second polarizers, due to the orthogonality of the two polarizing axes. Only the GMR, which experiences a change in its polarization axis upon interaction with the PCS, is able to pass the second polarizer, which is due to a filter effect and not a polarization rotation [44]. As seen in Equation (1), the resonance depends on the angle of incidence of the light. In order to minimize angular effects, a pinhole of 300 µm in diameter was used as a point light source for collimation. We used a lens with a focal length of 75 mm. The angular component of the collimated light was calculated to be 3 mrad and is therefore negligible. The camera detects the light coming from the PCS surface and enables spatial resolution. We split the image into its blue, green, and red subimages and evaluated only the red channel, as the resonance lies at the maximum of the spectral response of the red channel. Via surface chemistry, we introduced immobilized capturing agents to the surface of the PCS to which the biomarkers bind. The setup is usable in transmission as well as reflection mode.

The concept of transducing the resonance shift into an observable intensity shift is shown in Figure 1b. The resonance of the PCS lies on the falling shoulder of the LED spectrum (OSA 440 PY, OSA Opto Light, Berlin, Germany). We chose a cut-off filter (FESH0600, Thorlabs, Bergkirchen, Germany) with its edge on the falling shoulder of the LED spectrum in order to transduce any changes induced by refractive index changes into a decrease of the detected intensity. The combination of the LED spectrum and the cut-off filter leads to a narrowband sampling of the GMR. Any binding event leads to local changes of the refractive index and the resonance shift was transduced into an intensity shift due to the system response. These intensity changes were detected by a camera (UI-3260CP-C-HQ, IDS Imaging Development Systems GmbH, Obersulm, Germany) to allow for spatial analysis. The signal detection required no additional postprocessing as it corresponded simply to the average intensity within a ROI. All measurements shown here are linearly compensated for temperature drifts. Thermic effects are a challenge for any type of measurement [46]. However, the spatial resolution allows on-chip compensation [47,48,49,50]. The camera chip has a dynamic range of 255 a.u. and a photograph of the setup is shown in Figure 1c.

### 2.2. Fabrication of Photonic Crystal Slabs and Surface Functionalization

The fabrication of the PCS and the surface functionalization was conducted as explained previously [31,47,48,51,52,53]. Here, we used a PCS with a period length of 370 nm and a depth of 45 nm. Using a 370 nm period length allows design of TE and TM modes in the green to red region of the spectral camera response for which optical components are readily available. This reduces cost and enables a setup with off-the-shelf components. As a waveguiding layer we used titanium dioxide. We used 45 nm as a grating depth, as it provides a good tradeoff between Q factor and scattered light.

As immobilized capturing entities we used GST protein (custom product, Novatec Immundiagnostica GmbH, Dietzenbach, Germany), BSA biotin (A8549, Sigma-Aldrich, Taufkirchen, Germany), and protein A/G (21186, ThermoFisher, Bremen, Germany). GST protein and its corresponding antibodies are an inexpensive and easily available interaction pair, since they are used as purification tags [54]. Biotin and streptavidin are a well-established binding model for investigation of simplified biosensors, since they have a strong binding affinity [55]. Protein A/G is a recombinant protein which binds any type of immunoglobulin G (IgG) antibodies with high affinity and immunoglobulin A (IgA) and M (IgM) with low affinity [56,57]. Protein A/G allows use of biological samples such as unpurified whole blood as a simplified model for biosensing from samples of biological origin, due to its high affinity.

### 2.3. Fabrication of Microfluidics Based on Poly(dimethylsiloxane)

**Figure 2:**
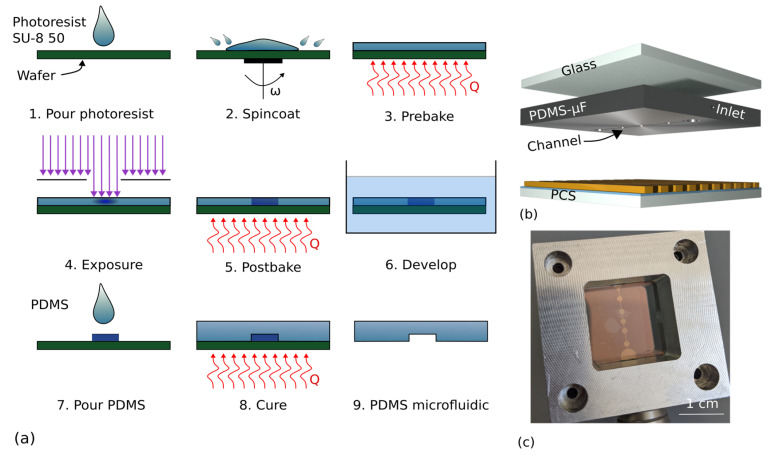
(**a**) Fabrication workflow of the poly(dimethylsiloxane) (PDMS) microfluidic (µF). (**b**) The concept of the microfluidic biochip. (**c**) An image of the microfluidic biochip mounted for measurement.

We combined microfluidics based on poly(dimethylsiloxane) (PDMS) with PCS in order to obtain low-cost microfluidic biochips. PDMS is known to be a good molding material with high malleability [58,59]. We used a two-step process. The first step involved the fabrication of the mold via a lithographic process on a 6-inch silicon wafer. The second part of the process used the mold to form microfluidic channels. The process is shown in Figure 2a.

First, the wafer was cleaned for 10 min in acetone and isopropanol and its surface was activated by exposing the wafer for 3 min to a plasma with 8 sccm and a power of 300 W (SI100, Sentech, Berlin, Germany). Then, the negative photoresist (SU-8 50, micro resist, Berlin, Germany) was poured onto the wafer and spincoated. Following that, the resist was pre-baked for 6 min at 65 °C and 20 min at 95 °C. A mask with the microfluidic layout was placed onto the resist in contact mode with subsequent UV light exposure. The exposure dose was 300 mJ/cm^2^. After curing, the resist needed to be post-baked. For this, the wafer was exposed for 1 min to 65 °C and then for 4 min to 95 °C. Following that, we developed the resist for 6 min in the corresponding developer. Finally, we dried the surface with nitrogen. The wafer then contained a surface relief, which was used as a mold. The heights of the structures were measured (VK-X260-K, Keyence, Neu-Isenburg, Germany) and were found to range between 45 µm to 60 µm.

The PDMS (Sylgard 181, Dow, Midland, USA) was mixed with its curing agent at a ratio of 8:1 and poured onto the mold containing the wafer. The PDMS was baked for 30 min at 130 °C. After cooling, the PDMS was peeled off the wafer and cut into pieces of size 25 mm × 25 mm. With a biopsy punch we added waste reservoirs and injection channels to permit application of the liquids. The transport of the liquids was enabled by degassing the microfluidic biochip in a vacuum prior to the measurement, as reported by Dimov et al. [60] and Yeh et al. [61]. By applying a liquid to the channel, the channel geometry is sealed off and the liquid is sucked in as the air within the channel reenters the PDMS, therefore creating negative pressure within the channel. This pressure difference drives the transport of the liquid autonomously without the need for external pumps. Combining this with a vacuum-sealed packaging could allow for single-use biochips with autonomous transport of liquids. The concept and an image of the mounted microfluidic biochip are shown in Figure 2b,c.

## 3. Results

### 3.1. Effects of the Cut-Off Filter on the Limit of Detection

**Figure 3:**
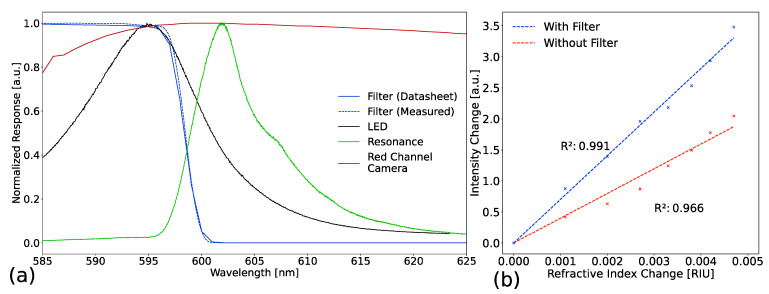
(**a**) Spectral response of the employed optical components. The four overlaying spectra depict the behavior of the cut-off filter, the light emitting diode (LED) spectrum, the guided-mode resonance (GMR) of the photonic crystal slab, and the red channel response of the camera. The plots are normalized for easier visual understanding. The intersection of the LED, transmission behavior of the filter, and the red channel camera response form the system response. The dashed blue line shows the measured filter behavior. (**b**) Detected intensity changes from a refractive index sweep performed with and without a filter. The changes are shown as positive to facilitate visual perception.

**Figure 4:**
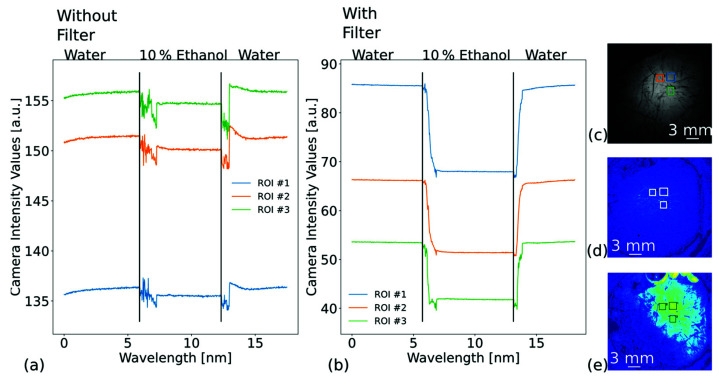
Effect of the cut-off filter on the sensitivity of the intensity-based camera setup (IBCS). The measurements were conducted consecutively, without changing the photonic crystal slab orientation. The cut-off filter was simply introduced. The setup was used in transmission. The regions of interest (ROIs) are the same for both experiments. The camera intensity values range from 0 to 255. (**a**) Red channel intensity change for a photonic crystal slab without a cut-off filter. (**b**) Red channel intensity change for a photonic crystal slab with the cut-off filter introduced. (**c**) Sample image with the corresponding ROIs. (**d**) Logarithmically enhanced difference image during exposure to 10% ethanol without the cut-off filter. (**e**) Same as (**d**) but with the cut-off filter inserted.

First, we explain the underlying concept, which is shown in Figure 3a,b. Figure 3a portrays the normalized response of the optical components. The red channel camera response is almost constant for the spectral range above 595 nm. It therefore has a negligible effect on the system response in this case. Without the filter, the LED would excite almost the entire GMR, since the resonance lies within the spectrum of the LED. A resonant shift due to refractive index change is therefore modelled by the LED spectrum (refer to Jahns et al. [31]). The filter changes the system response. The sharp edge of the cut-off filter introduces a more sensitive system response, as shown below in Figure 3b and Figure 4. The filter edge lies on the falling shoulder of the LED spectrum in order to transduce any changes of resonance position into a decrease of the camera intensity values. Furthermore, the edge of the cut-off filter needs to intersect the rising edge of the GMR. This ensures that any resonance shift due to refractive index change is transduced into a reduced signal. In addition, the rising shoulder of the GMR is almost linear and steeper, which benefits the sensitivity. However, the integration time needs to be increased as we cut off the light. We further show that the measured behavior (Agilent Cary 5000) of the filter fits the datasheet well.

For measurement, the PCS was placed in a fluid chamber, which consisted of the PCS, a gasket, and a second glass. The inflow and outflow of the liquids was realized by injecting wingflow cannulas through the gasket. The inflow was connected to a syringe containing the liquids, and the outflow was connected to a waste container. The volume of the fluid chamber was approximately 1.2 mL. For a thorough exchange we added 3 mL of liquid. This setup has been described in publications by our workgroup [31,47,48]. The performance effect exerted by the cut-off filter was analyzed by performing a refractometric sweep and step. Using refractive index sweeps in order to detect the bulk refractive index sensitivity of a PCS is a well-established proxy to model general ability for biosensing. A refractive index sweep is a successive change of liquids with different refractive indices in order to calculate the sensitivity of the PCS to a bulk refractive index change. This acts as a proxy for the performance of the PCS in a biosensing experiment. However, one has to consider that, for biosensing, the surface sensitivity is more important and that bulk sensitivity does not necessarily model this behavior sufficiently [20,21,35].

In Figure 3b we performed a refractive index sweep for a PCS with and without the cut-off filter in order to investigate the linearity of the system. The refractive index changes were made by creating glycerin solution from 1 wt% to 4 wt% in 0.5 wt% steps. The setup was used in transmission mode and the measurements were performed consecutively, meaning that the sweep was performed first without the filter and then with the filter, while the sample position was not altered at any point during the measurement. In both cases, the system responded linearly, while the slope with the cut-off filter was steeper than without the filter. We assume linear behavior to model the LOD for the data shown in Figure 4. The changes of detected intensity are shown as positive values, to facilitate visual perception of the behavior. A further analysis of how the data were calculated is given in Appendix A.

The effects of the filter on the performance of the IBCS are further investigated in Figure 4 and in Table 1. As before, the results for both plots were taken consecutively, meaning that after the measurement without the cut-off filter was finished, the filter was mounted into the optical path and the measurement was repeated. Then, the PCS was evaluated at the same ROIs, as shown in Figure 4c. The measurement was done in transmission. The refractometric step was performed by changing the content of the fluid chamber from water to a mixture of 10% ethanol in water and back to water. The refractive index change was measured (DR-201-95, Krüss, Hamburg, Germany) to be 5.0 × 10^−3^ RIU. Figure 4a,b show the camera intensity values, which range from 0 to 255. The results for the measurement without the cut-off filter are shown in Figure 4a. The change in signal was calculated by averaging the intensity value within the ROIs. When exposing the PCS to 10% ethanol, a signal change of approximately (1.1 ± 0.2) a.u. was detected for the three ROIs. The corresponding logarithmically enhanced difference image is shown in Figure 4d. With the cut-off filter, a strong increase in sensitivity was detected. These results are shown in Figure 4b,e. The observed intensity change increased on average to (14.7 ± 2.4) a.u. when exposed to 10% ethanol. Yet, due to the filter, the shutter time was increased from 10 ms to 140 ms. The detected sensitivity increased from (220 ± 50) a.u./RIU to (2940 ± 490) a.u./RIU. In addition, the triple standard deviation decreased slightly from (0.14 ± 0.01) a.u. to (0.09 ± 0.01) a.u. We attribute this to the cut-off filter that filtered out residual background light as well as the longer integration time. This led to a 22-fold improvement of the LOD from (7.1 ± 1.3) × 10^−4^ RIU to (3.2 ± 0.7) × 10^−5^ RIU for the evaluated ROIs. A single sample step was used, since the refractive index sensing was modelled to be quite linear as we have shown above and was also shown, for example, by Lin et al. [29] and Kenaan et al. [30]. However, Bläsi et al. [48] showed that the shape of the resonance influences the linearity of the response. Linearity is only a heuristic approach for a certain refractive index interval. The maximum refractive index step of 5.0 × 10^−3^ applied here lies within the linear range of the PCS. Moreover, we emphasize the effect the cut-off filter has on the order of magnitude of the LOD, which is already visible by the single-step comparison. In addition, the LOD is also affected by the ROI size, as shown below.

The strong increase of signal is also visible in the logarithmically enhanced image shown in Figure 4e. Furthermore, it should be noted that the order of the absolute intensity values changes once the cut-off filter is inserted. We attribute this to the inhomogeneous distribution of the GMR on the surface of a PCS [62]. This arises due to inhomogeneous sputtering, since the sample is not rotated during the process. This leads to thickness variations, which in turn alter the resonance wavelength at different loci of the PCS.

**Figure 5:**
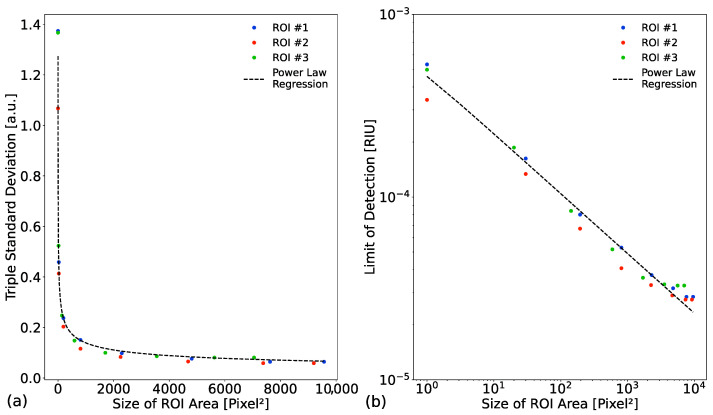
Effect of the region of interest (ROI) size on the noise and achievable limit of detection (LOD) in an intensity-based camera setup (IBCS) with cut-off filter using photonic crystal slabs (PCS). (**a**) The observed triple standard deviation with respect to the ROI size. It follows a power law distribution. (**b**) The corresponding achievable LOD for different ROI size from (**a**). The axes are logarithmically scaled leading to a linear representation of the power law.

As mentioned above, the advantage of this setup is that little postprocessing is necessary, as the intensity values of the camera image are directly averaged within the ROIs. However, the size of the ROI influences the noise level, as each pixel functions as a sample point. By increasing the ROI size, the noise is decreased and in turn the LOD is increased. This behavior is shown in Figure 5. In Figure 5a, we illustrate the observed triple standard deviation for different ROI sizes. The distribution of the triple standard deviation follows a power law distribution. As the noise directly influences the LOD, we plotted the obtained LOD for different ROI sizes in Figure 4b. By changing the scale for the x and y axis to a logarithmic scale, the power law is displayedas a power law regression line, which facilitates reading.. The figure shows that with increasing ROI size the LOD also improved. At greater ROI sizes, the obtained LOD differs more from the power law regression line. This is due to the inhomogeneous sensitivities of the PCS surface [62]. This behavior should be considered when designing a microfluidic layout for a camera-based detection setup. Here, for an LOD below 10^−4^, a ROI of at least 100 pixels should be taken.

### 3.2. Refractive Index and Biomolecular Sensing with a Microfluidic Biochip

**Figure 6:**
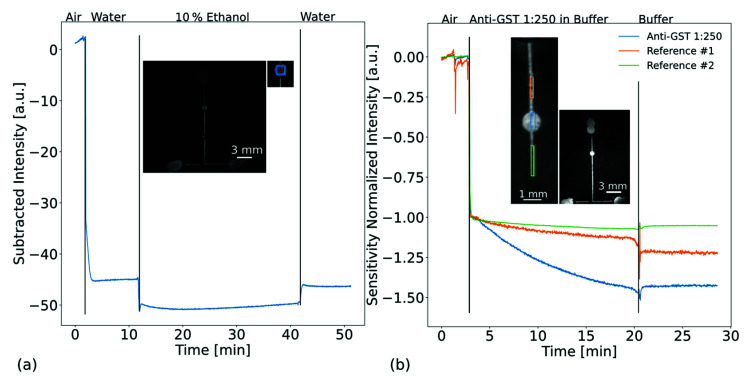
Recorded intensity changes of a microfluidic photonic-crystal biochip. The measurements were performed in transmission. (**a**) Refractometric measurements with a microfluidic biochip. The photonic crystal slab and the microfluidic were sealed off with an additional glass (refer to Figure 2b). The liquids were autonomously sucked into the microfluidic due to prior degassing of the PDMS. The photonic crystal slab was exposed to air, water, 10% ethanol–water and then water again. The refractive index change between water and 10% ethanol–water is 5.0 × 10^−3^ RIU. We attribute the remaining offset between the water exposure levels due to mixing of water and ethanol at the entrance of the microfluidic. (**b**) Biosensing with a microfluidic photonic-crystal biochip. The binding of glutathione S-transferase (GST) antibodies to immobilized GST protein was detected. The antigens are immobilized in the center chamber. The buffer containing the GST antibodies (diluted by 1:250) is sucked in autonomously, as the PDMS was degassed prior to usage. The images illustrate the regions of interest (ROI) where the intensity was evaluated and show a photograph of the sample in transmission before it was filled with liquid.

Similar to before, the performance of the microfluidic biochip was evaluated by performing a refractive index step. For this, a microfluidic biochip with one chamber was used. The setup is shown in Figure 6a. The measurement was performed in transmission.

First, we used the degassing method to assess the ability to transport liquids. For this, we stored the microfluidic biochip for 30 min in a vacuum. The vacuum was supplied via the inhouse system. A video of this experiment is shown in the Supplementary Information Video S1. After two minutes in air, we added 10 µL of water at the entrance of the microfluidic. Due to the high change in refractive index of the covering media from air to water, a strong change in intensity was detected when water was sucked in. After approximately 10 min, we added a 10% ethanol mixture and after another 30 min we added water again. We calculate the LOD to be 1.4 × 10^−4^ RIU.

Furthermore, this setup shows that the microfluidic is able to sustain a liquid transport for more than 30 min at ambient conditions. This is in accordance with other findings and showcases that this microfluidic biochip could be used in a non-laboratory setting [60,61]. For instance, Yeh et al. [61] have shown that by prepackaging under vacuum a single disposable chip can be obtained. Due to the packaging, the vacuum and thus the ability of liquid transport is maintained. The hardware requirements of the system introduced above are moderate, which renders the design of a point-of-care system for out-of-laboratory settings feasible. Combining these two concepts and by functionalizing the surface, an application under non-laboratory conditions as a single-use disposable in a point-of-care environment is imaginable.

In order to evaluate the ability of the setup for biosensing within a microfluidic biochip, we immobilized GST protein on the surface of the PCS. The results of this are shown in Figure 6b. A video of this measurement is shown in the Supplementary Information Video S2. For immobilization, we placed a 1 µL droplet of 100 µg/mL GST protein in the round chamber. After incubation, we passivated the rest of the surface with bovine serum albumin (BSA) (A2153, Sigma Aldrich, Taufkirchen, Germany). After degassing the microfluidic biochip for 30 min, we added GST antibodies diluted by a factor 1:250 in Dubelcco’s phosphate buffered saline (DPBS) (D8537, Sigma-Aldrich, Taufkirchen, Germany) at the entrance of the microfluidic channel. It should be noted that the manufacturer of the antibodies does not provide a defined concentration. They state a range of 8–15 mg/mL, as the product is a polyclonal antibody. As we cannot calculate a specific concentration, we simply state a dilution factor. Furthermore, we diluted the sample as we wanted to analyze the behavior in a less concentrated sample to obtain slower reaction kinetics as an initial step. We evaluated the intensity changes for three ROIs and normalized the sensitivity to the induced refractive index step from air to DPBS. This compensates local sensitivity dependencies and allows the signals to be compared. Figure 6b shows that a change of intensity in the shape of a biological binding curve was detected in the area where GST proteins were immobilized [63]. This behavior was also visible for the two reference ROIs, although it was a lot less prominent. This indicates unspecific binding to the passivated surface and is a common phenomenon in biosensing. Introducing polyethylenglycols (PEG) to surfaces is a promising approach to suppress unspecific binding in biosensors [64]. Recently, we have shown that it is possible to immobilize aptamers to functionalized surfaces containing PEG molecules and aim to use this functionalization route for future biosensors [65]. After approximately 17 min, we applied 10 µL of pure DPBS. This stopped the binding behavior of the GST antibodies, indicating that a true binding of GST antibodies was detected.

As initially stated, the advantage of the system lies in its spatial resolution. It allows visual confirmation of binding via the circular appearance of intensity changes and the use of reference regions to compensate background behavior. This is additional information and helps in understanding the observed behavior. In order to be able to visually investigate the behavior, we used a microfluidic photonic-crystal biochip with a width of 7 mm and immobilized two spots of BSA biotin on the PCS. The immobilization was achieved by placing two 1 µL spots with a BSA biotin concentration of 100 µg/mL. Again, we passivated the remaining surface with BSA after incubation of the capturing spots.

**Figure 7:**
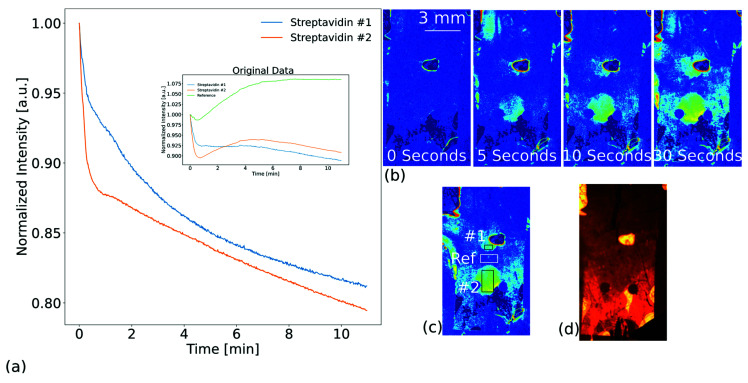
Results of sensing streptavidin binding to BSA biotin immobilized to the surface of the photonic crystal slab (PCS). Measurement was done in reflection. Biotin was immobilized where the ROI for #1 and #2 was positioned. (**a**) Detected intensity changes after exposure to a streptavidin concentration of 2.9 µg/mL for three regions of interest. The two plots are divided by the reference line, which is visible in the inlet. (**b**) An image series of logarithmically enhanced difference images showing the binding effect within the first 30 s. (**c**) Logarithmically enhanced difference images indicating the sensing and referencing regions. (**d**) A photograph of the microfluidic biochip under LED illumination. A scattering entity is brightly visible in the area of functionalization spot #1 and prevents detection in this area.

The results are shown in Figure 7. The measurement was performed in reflection. Differing from the previous measurements, we used wingflow cannulas for the inlet and outlet of the liquid. The chamber was initially filled with DPBS and we injected a concentration of 2.9 µg/mL streptavidin diluted in buffer. The intensity change was recorded in three ROIs and is shown in Figure 7a. The inset displays the normalized data for the three ROIs individually. Unfortunately, there was a scattering entity brightly visible in the area of functionalization spot #1 that prevented full detection underneath. Two ROIs were chosen for evaluating the binding of streptavidin and one for referencing, as is visible in Figure 7c. The two curves of the main plot were obtained by dividing them by the reference signal. The two streptavidin ROIs showed a large change of intensity directly after exposure to streptavidin. This is an expected behavior. However, a background drift was also observed, which is shown by the green referencing line. Without the additional visual information, the understanding of the behavior would be impaired. Yet, using the spatial information, the signals of the streptavidin ROIs are attributed to true binding. This is underlined by the time series shown in Figure 7b. A video containing the binding event of the logarithmically enhanced difference images is provided in the Supplementary Information Video S3. The two circular binding spots of immobilized BSA-biotin are clearly visible. This was not observed for the referencing ROI. Further, it can be seen that the signal continued to decrease once the referencing area reached a plateau. This was approximately 5 min after streptavidin exposure. This shows that visual information is an aid in understanding the detected behavior and is an advantage of the detection principle.

### 3.3. Biosensing of Immunoglobulins G from Unpurified Whole Blood

**Figure 8:**
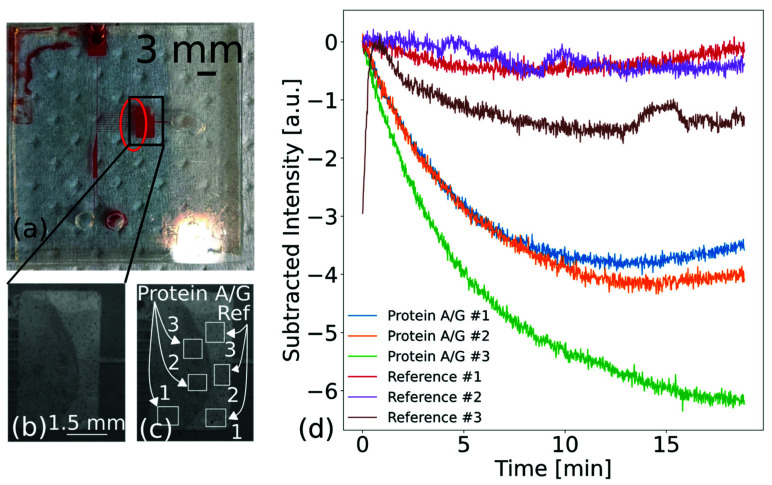
Microfluidic photonic-crystal biochip for immunoglobulin G (IgG) detection from unpurified whole blood. The oval indicates the approximate spotting area of protein A/G. A video of the inflow is shown in the Supplementary Information Video S6. (**a**) A photograph of the microfluidic biochip after exposure to blood. All channels are filled with blood. The red oval indicates the approximate position of the immobilized protein A/G. (**b**) Images of the chamber after binding. A clear contour line where binding took place is visible. (**c**) Positioning of the three sensing and referencing ROIs. (**d**) Recorded intensity changes for three sensing and three referencing ROIs within a microfluidic chamber. They are all subtracted by their 90 s value in order to position them on the same level for easier understanding.

We investigated the ability of the microfluidic biochip combined with an IBCS to sense IgG from unpurified whole blood. The blood was obtained from the University Hospital Schleswig-Holstein (UKSH). Blood samples were provided exclusively by healthy volunteer donors from our research group. The samples were used immediately without prior storage or cooling. In order to suppress coagulation, blood was withdrawn with EDTA or heparin as an anticoagulent. As an immobilization agent, 1 µL of 100 µg/mL protein A/G was placed on the surface of the PCS. After incubation, the surrounding area was again passivated with BSA. A microfluidic designed on principles of the ‘Zweifach–Fung’ effect was used [66,67]. This effect has been proved capable of continuously separating cell constituents from whole blood. We aimed for this effect in order to mitigate the disturbance of solid constituents of the blood during the measurement. Additionally, we performed this measurement in reflection, since blood is an opaque liquid. A measurement in transmission would not be possible, as the light would be attenuated by the opaqueness and the constituents of the blood (refer to Supplementary Information Videos S4 and S5). We again used the degassing method as the force for the transport of the blood. A video of the transport is provided in the Supplementary Information Video S6. The blood needed about 90 s to fill the sensing chamber almost completely. The results of the measurement are shown in Figure 8. In Figure 8a, the biochip layout is visible. The blood is placed at the top and is transported via the channel to the chamber in the center and bottom of the microfluidic. The central chamber covers approximately half of the immobilization spots, which is indicated by the red oval. Investigating the image after 90 s of whole blood exposure, a contour of a half-circle emerged, indicating the binding of IgG to protein A/G, as is visible in Figure 8b. Figure 8c,d show the position of the sensing and referencing ROIs and the detected signal, respectively. The signal was zeroed at 90 s once the blood had completely filled the chamber. Contrasting the sensing and referencing ROIs, a change in signal behavior was detected. It can be seen that the three sensing ROIs exhibited changes in intensity. The three referencing ROIs, however, did not exhibit this behavior. The strong contrast suggests that binding took place where protein A/G was immobilized. The rather moderate intensity changes of approximately 3.5 a.u. shown here stem from the fact that the majority of the signal change occurs within a few seconds after exposure to whole blood [47]. This is shown in the Supplementary Information Videos S6 and S7. However, we show the signals after 90 s, to permit comparison of different ROIs within the chamber. Combining the visual information with the intensity information corroborates that binding took place.

The filtering effect of the microfluidic is shown in Figure 8a. It can be seen that the filtering did not work as intended, since the chamber completely filled with blood. We attribute this to insufficient speed in the vertical main channel, as the liquid was not transported by an external pump. However, this shows that the setup is able to detected biomarkers from unpurified whole blood within a microfluidic biochip. Using whole blood as the sample is a challenge, since it is a highly complex fluid. While label-free sensing from whole blood has been shown [68], no works based on PCS (apart our work in [47]) regarding this matter have been reported. Jahns et al. [69] used a filter to rid the whole blood of its solid constituents. Here, we demonstrated the ability of the system to sense directly from whole blood without any preprocessing [70]. This is of great interest, as filtering may lead to hemolysis, which affects the composition of the blood and is a major issue of concern in clinical fields [58]. Further, almost no fouling nor unspecific binding was detected. We suppose that the reduced amount of solid constituents and the constant flow due to the microfluidic mitigates these effects. Previously, we have shown that fouling effects in a confined chamber need to be considered when working with unpurified whole blood [47]. This is also shown in Supplementary Information Video S7. The results obtained here suggest that using a microfluidic might mitigate this problem.

## 4. Conclusions

In this work we have introduced an intensity-based camera setup for narrowband sampling of a GMR by employing a sharp-edged cut-off filter. By employing the cut-off filter, we reduced the intensity and could increase the shutter time without saturating the camera. The cut-off and longer integration led to a slight decrease of the detected noise from (0.14 ± 0.01) a.u. to (0.09 ± 0.01) a.u. More importantly, the setup showed a 13-fold improvement in the sensitivity from (220 ± 50) a.u./RIU to (2940 ± 490) a.u./RIU. This led to a 22-fold improvement of the LOD from (7.0 ± 1.3) × 10^−4^ RIU to (3.2 ± 0.7) × 10^−5^ RIU when the cut-off filter was introduced. Furthermore, we showed that the size of the ROI strongly influences the achievable noise and subsequently the LOD, since each pixel acts as a sampling point. Our LOD is comparable to previously published findings [21,29,39,40]. However, no additional postprocessing is necessary in our setup, since the signal change corresponds to the average intensity within an ROI. This renders this detection method more robust, as no a posteriori knowledge is needed for the analysis.

We further investigated the ability of the setup to perform the readout of a microfluidic photonic-crystal biochip. We used autonomous transport of liquids based on a negative pressure method due to degassing of the PDMS. We were able to maintain transport of liquids within the biochip for more than 30 min. This is a sufficient length for a possible single-use consumable. We illustrated the biosensing ability by detecting GST antibodies diluted by a factor of 1:250 with a microfluidic biochip, as well as streptavidin at a concentration of 2.9 µg/mL in transmission and reflection, respectively. We have shown the advantage of including additional visual binding information, which enables visual confirmation of binding behavior and use of on-chip information for background compensation. Furthermore, we have shown for the first time the direct sensing of IgG from unpurified whole blood with a microfluidic photonic-crystal biochip with autonomous transport of blood. This is of high interest for potential use in a point-of-care system. Overall, we were able to detect biomolecular binding within less than 5 min of exposure to the sample. In addition, the amount of necessary equipment is moderate, rendering this approach highly interesting for other research as well as commercialization.

## Figures and Tables

**Table 1:** Analysis of the cut-off filter effect on the performance parameters of the intensity-based camera setup. Limit of detection is abbreviated by LOD. The arbitrary units are indicative of the camera intensity values, which range from 0 to 255.

Cut-Off Filter	Shutter Time [ms]	Triple Standard Deviation [a.u.]	Signal Difference [a.u.]	Sensitivity [a.u./RIU]	LOD [RIU]
Without filter	10	0.14 ± 0.01	1.1 ± 0.2	220 ± 50	(7.1 ± 1.3) × 10^−4^
With filter	140	0.09 ± 0.01	14.7 ± 2.4	2940 ± 490	(3.2 ± 0.7) × 10^−5^

**Figure A1:**
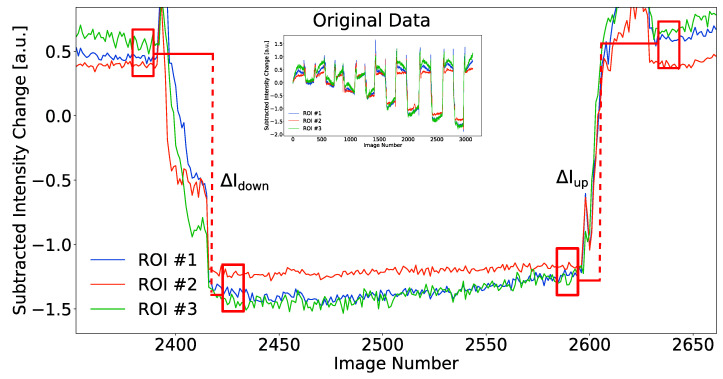
Evaluation concept for the refractive index sweep shown in Figure 3b. At each glycerin step, the average step height of the three ROIs is evaluated by calculating the mean of five sample points before and after the refractive index change. This enables drift compensation, which is visible in the original data inlet. The step height is averaged by adding the two obtained values and then halving the result.

## Data Availability

The data are currently not available to the public, but may be supplied upon reasonable request.

## Supplementary Materials

The following supporting information can be downloaded at: https://www.mdpi.com/article/10.3390/bios13070687/s1, Video S1: S1_Refractometric_Measurement_PCS_with_MF, Video S2: S2_Biosensing_AntiGST_PCS_with_MF, Video S3: S3_Biosensing_Streptavidin_PCS_with_wide_MF, Video S4: S4_constituents_of_blood_in_microfluidic, Video S5: S5_autonmous_blood_flow_in_microfluidics, Video S6: S6_Biosensing_IgG_PCS_with_MF, Video S7: S7_Biosensing_IgG_PCS_with_Whole_Chamber.

## Appendix A

The results shown in Figure 3b were obtained by performing two consecutive refractive index sweeps. For this, an array of glycerin solutions starting from 1 wt% to 4 wt% in 0.5 wt% steps was fabricated. The refractive indices were measured with the DR-201-95 refractometer from Krüss GmbH. The fluid chamber was then iteratively flushed with water, then exposed to a glycerin concentration and then back to water. The exposure durations were approximately 180 s. The change in intensity was calculated by averaging five values shortly before and shortly after the refractive index change, respectively. The step height was calculated twice for each refractive index step, as the measurement enables the evaluation from water to the glycerin step and back again. This allowed compensation for the existing drift. The obtained results for the no-filter measurement are shown in the inset of Figure A1. In the main figure, the concept of the step height calculation is portrayed. The data obtained for the filter measurement were evaluated in the same way.

As a concluding remark, we want to point out that the measurements shown in Figure 4 and Figure 3b and Figure A1 were all performed with the same PCS. However, there was a time period of more than a year between the former and the latter two. The PCS was used extensively within this time frame for research purposes, which deteriorated its performance. This led to the less prominent increase of sensitivity via the use of a cut-off filter, as is visible in Figure 3b.
